# Incorporation of Second-Tier Biomarker Testing Improves the Specificity of Newborn Screening for Mucopolysaccharidosis Type I

**DOI:** 10.3390/ijns6010010

**Published:** 2020-02-07

**Authors:** Dawn S. Peck, Jean M. Lacey, Amy L. White, Gisele Pino, April L. Studinski, Rachel Fisher, Ayesha Ahmad, Linda Spencer, Sarah Viall, Natalie Shallow, Amy Siemon, J. Austin Hamm, Brianna K. Murray, Kelly L. Jones, Dimitar Gavrilov, Devin Oglesbee, Kimiyo Raymond, Dietrich Matern, Piero Rinaldo, Silvia Tortorelli

**Affiliations:** 1 Biochemical Genetics Laboratory, Department of Laboratory Medicine and Pathology, Mayo Clinic, Rochester, MN 55905, USA; lacey.jean@mayo.edu (J.M.L.); white.amy1@mayo.edu (A.L.W.); pino.gisele@mayo.edu (G.P.); studinski.april@mayo.edu (A.L.S.); gavrilov.dimitar@mayo.edu (D.G.); oglesbee.devin@mayo.edu (D.O.); raymond.kimiyo@mayo.edu (K.R.); matern@mayo.edu (D.M.); rinaldo@mayo.edu (P.R.); 2 Division of Pediatric Genetics, Metabolism and Genomic Medicine, Department of Pediatrics, University of Michigan, Ann Arbor, MI 48109, USA; firachel@med.umich.edu (R.F.); ayeshaah@med.umich.edu (A.A.); 3 Division of Genetic, Genomic and Metabolic Disorders, Children’s Hospital of Michigan, Detroit, MI 48201, USA; lspencer@dmc.org; 4 Rare Disease Institute, Children’s National Health System, Washington, DC 20010, USA; sviall@childrensnational.org; 5 Division of Medical Genetics and Genomic Medicine, Monroe Carell Jr. Children’s Hospital at Vanderbilt, Nashville, TN 37232, USA; natalie.shallow@vumc.org; 6 Division of Genetic and Genomic Medicine, Nationwide Children’s Hospital, Columbus, OH 43205, USA; amy.siemon@nationwidechildrens.org; 7 Pediatric Genetics, East Tennessee Children’s Hospital, Knoxville, TN 37916, USA; jahamm@etch.com; 8 Division of Medical Genetics and Metabolism, Children’s Hospital of the King’s Daughters, Norfolk, VA 23507, USA; brianna.murray@chkd.org (B.K.M.); kelly.jones@chkd.org (K.L.J.)

**Keywords:** newborn screening, MPS I, second-tier testing, GAGs, biomarkers, postanalytical interpretation

## Abstract

Enzyme-based newborn screening for Mucopolysaccharidosis type I (MPS I) has a high false-positive rate due to the prevalence of pseudodeficiency alleles, often resulting in unnecessary and costly follow up. The glycosaminoglycans (GAGs), dermatan sulfate (DS) and heparan sulfate (HS) are both substrates for α-l-iduronidase (IDUA). These GAGs are elevated in patients with MPS I and have been shown to be promising biomarkers for both primary and second-tier testing. Since February 2016, we have measured DS and HS in 1213 specimens submitted on infants at risk for MPS I based on newborn screening. Molecular correlation was available for 157 of the tested cases. Samples from infants with MPS I confirmed by *IDUA* molecular analysis all had significantly elevated levels of DS and HS compared to those with confirmed pseudodeficiency and/or heterozygosity. Analysis of our testing population and correlation with molecular results identified few discrepant outcomes and uncovered no evidence of false-negative cases. We have demonstrated that blood spot GAGs analysis accurately discriminates between patients with confirmed MPS I and false-positive cases due to pseudodeficiency or heterozygosity and increases the specificity of newborn screening for MPS I.

## 1. Introduction

Interest in newborn screening for Mucopolysaccharidosis type I (MPS I) has increased following the development of dried blood spot screening assays [1], availability of effective treatments [2,3], and increasing evidence that early intervention improves patient outcomes [4]. In early 2016, following systematic evidence-based review and subsequent approval from the Secretary of the Department of Health and Human Services, MPS I was added to the Recommended Universal Screening Panel. Numerous pilot and full-population screening programs using measurement of α-l-iduronidase (IDUA) activity have been underway in several countries and US states—many of which have experienced a high false-positive rate due to the overlap of IDUA activity between unaffected and affected patients and a high incidence of pseudodeficiency alleles in *IDUA*, resulting in overall poor specificity for MPS I [5,6,7]. While several programs have implemented second-tier molecular testing in an attempt to address the poor specificity of enzymatic screening alone, this approach has proved to be problematic due to the discovery of private mutations, variants of unknown significance, identification of carrier status and limitations of utilized methodology [8,9]. In addition, the high incidence of genotypic variants leading to pseudodeficiency of IDUA results in excessive performance of potentially unnecessary molecular genetic testing [10]. Pseudodeficiency for IDUA was first reported in 1985 [11], and was thought to be a rare occurrence prior to newborn screening.

Measurement of glycosaminoglycans (GAGs), in particular dermatan sulfate (DS) and heparan sulfate (HS), in dried blood spots by several methodologies has been used for the evaluation of at risk patients as well a primary newborn screen for MPS I [12,13,14]. With both prenatal evidence of GAG storage [15] and confirmation that DS and HS levels can accurately discriminate newborns with severe and attenuated forms of MPS I from unaffected newborns [12], implementation of biomarker testing in the newborn period is a logical next step to address the shortcomings experienced with enzyme screening and second-tier molecular testing. Our laboratory has developed a stepwise approach to newborn screening for MPS I by incorporating second-tier biomarker testing of the GAGs, DS and HS, following identification of decreased IDUA activity. Second-tier testing utilizes the original newborn screening sample, thereby eliminating recall of the infant for additional testing [16]. This model reduces the number of false-positive results and improves the specificity of newborn screening not only for MPS I [10] but also for other conditions [16]. Testing of dried blood spot GAGs can also be performed in the clinical setting for evaluation of screen-positive infants referred by the respective newborn screening program for possible MPS I. Dried blood spots are an ideal sample type to obtain from newborns, as urine collection can be difficult. Here, we report our experience with the incorporation of DS and HS measurement in the newborn screening process for MPS I. 

## 2. Materials and Methods

### 2.1. Analytical Methods and Reference Population

The biochemical method used for GAGs analysis in dried blood spots has previously been described [12,17]. Initial validation [18] showed a clear differentiation of known MPS I cases from cases carrying pseudodeficiency alleles. Reference values are as follows: DS: Newborn ≤ 2 weeks: ≤ 200 nmol/L; >2 weeks: ≤ 130 nmol/L. HS: Newborn ≤ 2 weeks: ≤ 96 nmol/L; >2 weeks: ≤ 95 nmol/L.

### 2.2. Study Population

Between February 2016 and August 2019, we tested 1213 specimens, belonging to 1166 newborns (639 males and 527 females) from birth through 6 weeks of age that showed low IDUA activity as a result of newborn screening for MPS I in their state. Forty five newborns were tested two or more times (repeat newborn screening sample for different reasons), accounting for the discrepancy of tested specimens. Testing was performed as either second-tier testing on the DBS collected at birth as part of the newborn screening process (*n* = 1019 specimens, *n* = 973 newborns) by three states (median age of collection 23.98 h; min 5 h, max 1151 h), or as a component of follow up to a presumptive positive newborn screening result for MPS I (*n* = 194 specimens, *n* = 193 newborns; median age of collection 20 days; min 23.98 h, max 1151 h).

### 2.3. Confirmatory Testing

The results of additional confirmatory testing were available for 211 patients, including IDUA activity and/or molecular genetic analysis of *IDUA. IDUA* variant classification for internally analyzed cases was performed as per ACMG Guidelines [19]. Variant classification for outside testing was provided by clinician report.

We performed repeat enzyme activity either by fluorometry or MS/MS [20] in 162 (99 + 61 + 2 (both)) cases. The *IDUA* genotype was available for 157 cases. 

The study was approved by Institutional Review Boards either based on a local protocol or by acceptance of the protocol established at Mayo Clinic (IRB #15-005393; approved 3 September 2019) as the lead organization for this study.

### 2.4. Post-Analytical Interpretation

**Figure 1:**
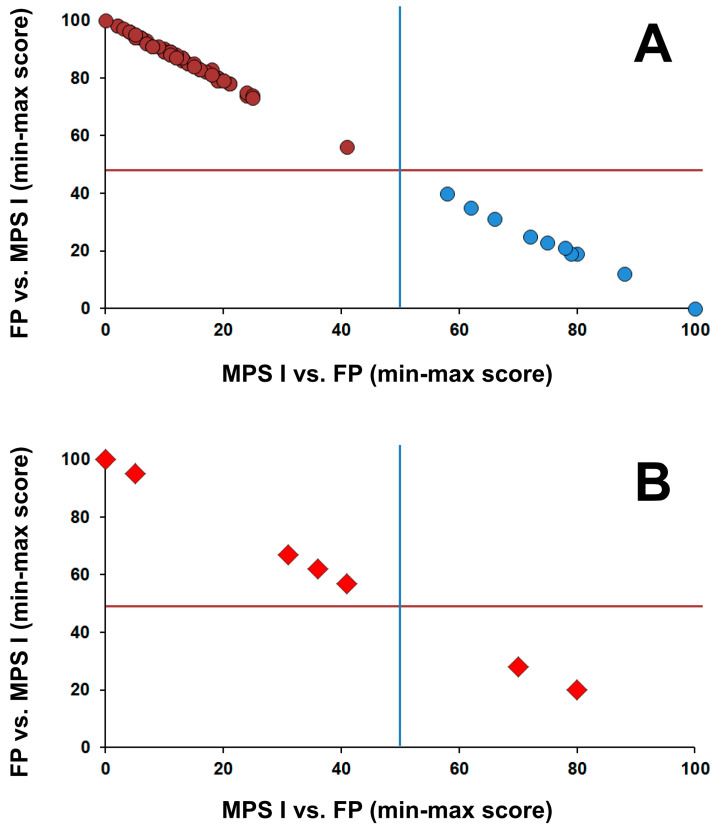
CLIR dual scatter plot; calculated to help determine the differential diagnosis between low IDUA activity more likely being true positive due to MPS I vs. false positive due to pseudo deficiency or *IDUA* carrier status. (**A**) Distribution of scores of cases (MPS I (blue dots) *n* = 10; false positives (purple circles) *n* = 67) confirmed by genotyping; (**B**) Distribution of scores of 7 cases with two variants in trans and at least one variant of uncertain significance (VUS; either pathogenic/VUS and VUS/VUS) and one case with no genotype.

A single condition tool for MPS I was created by the software Collaborative Laboratory Integrated Reports (CLIR, version 2.19; https://clir.mayo.edu). CLIR is a multivariate pattern recognition software and web application that maintains an interactive database of laboratory data from multiple sites and provides tools to be used as an aide to support result interpretation. CLIR was first applied to calculate likelihood of metabolic disorders linked to amino acids and acylcarnitines tested by tandem mass spectrometry [21,22]. When a patient triggers an informative score for MPS I, a dual scatter plot is then deployed to further classify the case as either MPS I or a false positive. This tool is based on the markers included in the second-tier test (HS, DS, and their calculated ratio). The output is a plot is divided in four quadrants by lines drawn at the midpoint between the lowest combined score of MPS I and the highest combined scores of false-positive cases (*x* axis, vertical line) and between the lowest combined score of false positives and the highest combined score of MPS I (*y* axis, horizontal line). Cases falling in the lower right quadrant (light blue circles; Figure 1A) are consistent with MPS I. Cases that fall in the upper left quadrant (purple circles; Figure 1A) are consistent with false-positive cases.

## 3. Results

Out of 1213 specimens, 1067 were found to have had normal concentrations of both DS and HS (group 1), 76 had either DS or HS above the reference value for age (group 2) and 23 were found to have concentrations of both DS and HS above the reference values for age (group 3). Characteristics of IDUA variants for all resolved cases are available in Table S1.

### 3.1. Patient Cohorts

#### 3.1.1. Group 1

The *IDUA* genotype was available for 121 (11.3%) of the 1067 cases in this group. Two had normal sequencing. Seventy four had confirmed pseudodeficiency, with pseudo variants identified on both alleles. Of these, 32 cases were found to be homozygous for the c.235G>A (p.A79T) variant, which has been previously reported to have an allele frequency of 4.05% in African Americans [7]. Thirty seven cases were compound heterozygous for a pathogenic variant or variant of unknown significance (VUS) and a pseudodeficiency or wild-type allele, suggestive of carrier status. Eight cases were compound heterozygous for a pathogenic, likely pathogenic, or VUS and a VUS. No cases were identified with two pathogenic or likely pathogenic variants. To our knowledge, no cases in this group had any clinical or additional biochemical evidence suggestive of MPS I.

#### 3.1.2. Group 2

The *IDUA* genotype was available for 13 (17%) of the 76 cases in this group. Pseudodeficiency variants were identified in all but two cases of group 2, either in both (*n* = 7) or in one (*n* = 4) allele. One case was compound heterozygotes for a pathogenic variant and a VUS, while the other had two VUS alleles. To our knowledge, no cases in this group had any clinical or additional biochemical evidence suggestive of MPS I.

#### 3.1.3. Group 3

**Figure 2:**
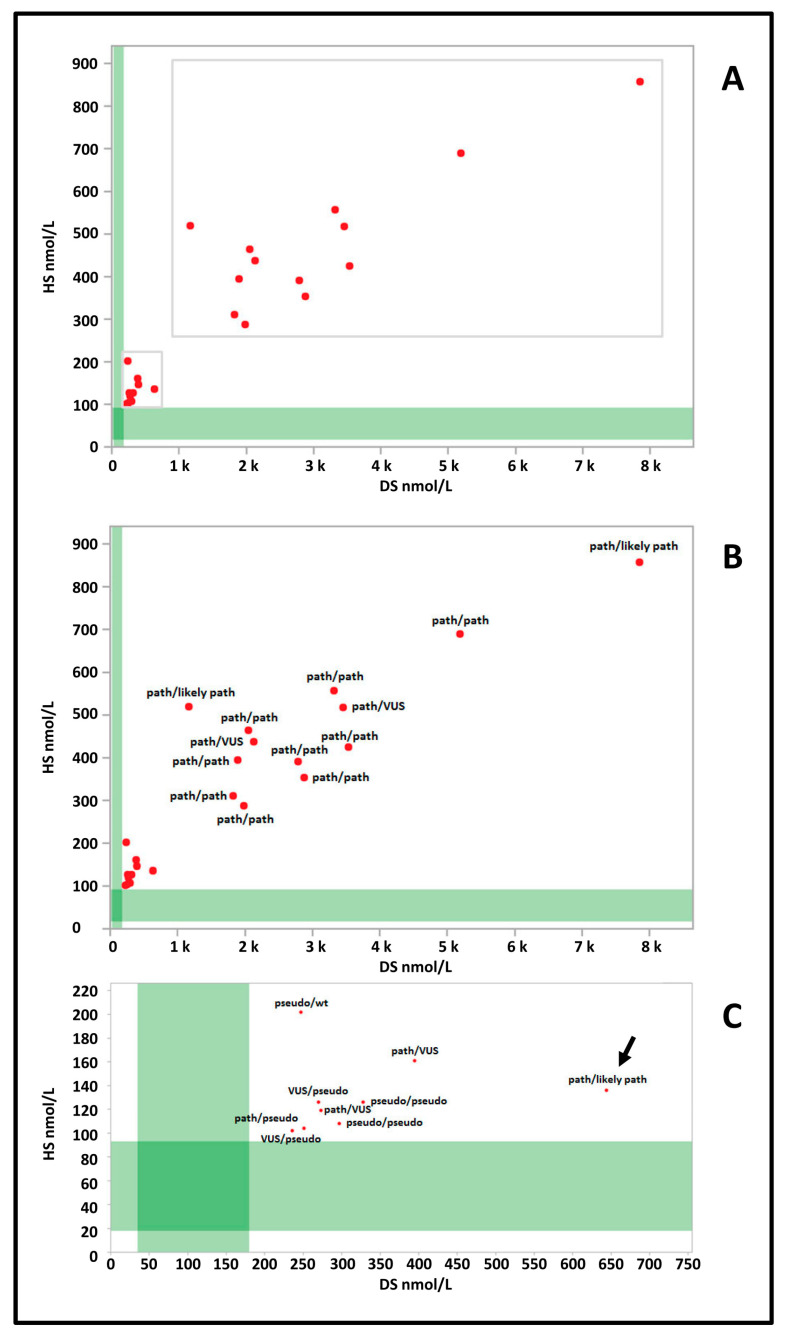
(**A**) Dermatan (DS) and heparan (HS) sulfate values of group 3 cases (red dots) are plotted against the reference population (green bands) (DS *n* = 3113; HS *n* = 3165). (**B**) *IDUA* genotypes of cases with higher levels of both DS and HS. (**C**) *IDUA* genotypes of cases with lower levels of DS and HS. path: pathogenic variant; likely path: likely pathogenic variant; VUS: variant of unknown significance; pseudo: pseudo deficiency allele; wt: wild type.

The *IDUA* genotype was available for 22 (96%) of the 23 cases in this group. One case was subsequently found to have normal IDUA activity on confirmatory testing and therefore molecular genetic analysis of *IDUA* was not performed per clinician discretion. Figure 2A shows how this group is distributed in two subgroups. Thirteen cases show a very high concentration of both DS (range: 1167–7859 nM; median 2795 nM) and HS (range 255–857 nM; median 432 nM). The most common pathogenic variant worldwide, c.1205G>A [23], was present in one allele in seven cases and was homozygous in three cases; only two alleles in two patients of this group were classified as VUS (Figure 2B).

The second subgroup was characterized by lower concentrations of DS (range: 236–644 nM; median 273 nM) and HS (range 102–202 nM; median 126 nM) (Figure 2C). Only one case (arrow Figure 1C) carried a pathogenic variant in compound heterozygosity with a likely pathogenic variant. Two were found to be homozygous for the c.235G>A (p.A79T) variant and two had one pathogenic variant associated with a VUS. The case with normal IDUA activity on confirmatory testing would have fallen in this subgroup.

### 3.2. Postanalytical Interpretation

The application of the dual scatter plot interpretive tool (Figure 1A) to two cases in group 2 and five cases in group 3 with two variants (with at least one being a variant of uncertain significance), and one case in group 3 with no genotyping allowed classification of two of these cases as MPS I and the remaining as false positive (Figure 1B).

## 4. Discussion

Newborn screening programs that include lysosomal storage disorders utilizing a one-tiered, enzymatic approach require that all cases with decreased enzyme activity be referred for confirmatory testing and evaluation. Reports from several newborn screening programs utilizing this model suggest that the positive predictive value (PPV) for MPS I newborn screening using IDUA enzyme activity alone is approximately 3% [5,6]. Hopkins et al. [6] reports Missouri’s experience over 4 years of newborn screening for lysosomal storage disorders. During this time period, 133 infants out of 308,000 screened were referred for confirmatory testing and evaluation due to decreased IDUA activity. Out of those referred, 71 cases were attributed to pseudodeficiency, (1/4338) while only two cases were confirmed as MPS I (1/154,000). Similar results were reported by Burton et al. [5], with 151 infants out of 219,793 screened referred for confirmatory MPS I testing and an incidence of 1/7326 for IDUA pseudodeficiency and 1/219,793 for MPS I. These findings are reproduced in several additional reports [24,25,26,27,28]. With the overall incidence of pseudodeficiency being approximately 16 times higher than true disease, newborn screening programs and clinicians involved in the evaluation of referred infants will continue to be overwhelmed with false-positive results if the enzymatic screen alone is utilized. 

Newborn screening programs incorporating second-tier molecular genetic testing also have poor specificity for MPS I and refer at risk babies based on decreased α-l-iduronidase levels alone, because molecular results may take days or weeks to return. Because of high referral numbers requiring second-tier molecular analysis during their testing pilot, the North Carolina newborn screening program incorporated postanalytical interpretation using Collaborative Laboratory Integrated Reports (CLIRs) into their testing algorithm, which provides on-demand web-based analysis using evidence-based segregation of laboratory results from affected patients vs. false-positive cases. Although this was shown to decrease the number of screen positive cases by 64%, only one confirmed case of MPS I was identified out of 19 cases referred for second-tier *IDUA* molecular testing [7]. 

Since February 2016, we tested 1213 specimens from cases identified as at risk for MPS I by newborn screening. Based on available follow-up data, we believe to have demonstrated that blood spot GAG analysis in either the initial newborn screening card (performed as a second-tier test) or clinically submitted specimens from infants with decreased IDUA activity identified by newborn screening accurately discriminates between patients with confirmed MPS I and false-positive cases due to *IDUA* pseudodeficiency or heterozygosity. All samples tested from infants confirmed by *IDUA* molecular analysis to have MPS I had significantly elevated levels of DS and HS compared to those with confirmed *IDUA* pseudodeficiency and/or heterozygosity. If newborn screening referrals were limited to patients identified with elevated DS and HS by our laboratory, only 23 patients would have actually required genotyping, which translates to a PPV of 74% (17/23). Accordingly, we have shown in a prospective cohort that blood spot GAG analysis and postanalytical interpretation can significantly increase the specificity of newborn screening for MPS I and should be incorporated into the screening process in order to improve overall program performance. Recently, Burlina et al. [29] reported that retrospective incorporation of second-tier GAG analysis in dried blood spots resulted in a PPV of 100% for MPS I, with elevated DS and HS in 2/26 cases with positive first-tier results.

Analysis of our testing population and correlation with molecular results identified few false-positive results and uncovered no evidence of false-negative cases at the time of manuscript submission. While several reports [12,13,14] demonstrate elevation of GAGs in newborns with MPS, there is little data on attenuated cases with respect to the timeline of GAGs accumulation and reliability of newborn screening for diagnosis. We acknowledge the possibility that newborns with attenuated MPS I may escape diagnosis using this screening algorithm, and therefore must rely on retrospective and prospective data collection as well as the exploration of alternative and possibly more sensitive testing methodologies [30] to address this limitation in the newborn screening setting. 

Our findings suggest that second-tier GAGs testing and postanalytical interpretation has several important benefits compared to molecular testing, such as avoidance of unnecessary patient recall, decreased assay cost and turnaround time, the prevention of identification of pseudodeficiency, carriers, and cases with inconclusive molecular results which may ultimately receive unnecessary clinical follow up, as well the provision of an actual biochemical phenotype of GAGs metabolism. As molecular analysis alone can be an unreliable tool for prediction of disease severity, GAG analysis combined with the use of postanalytical tools can help to determine pathogenicity of those dreaded variants of uncertain clinical significance. A recent publication by Clarke et al. [31] discusses the complexity of using genotypic information to predict phenotype in MPS I, with unique genotypes in 12.4% and 40% of patients with severe and attenuated MPS I, phenotypic variability associated with certain missense variants, and inability of current biochemical assessments to predict phenotype. It is also important to note that while the data originated from a voluntary patient registry, 18 patients had only one *IDUA* variant reported, highlighting the limitations of molecular testing without accompanying biochemical phenotyping as a component of newborn screening.

As there is evidence that GAGs accumulation in mucopolysaccharidoses occurs prenatally [15], measurement of DS and HS in newborn screening blood spots can be successfully applied in the newborn screening setting for conditions other than MPS I. Several states have recently added MPS II, also known as Hunter syndrome, to their testing panels and newborn screening has already been underway in Japan and Taiwan for several years. Both published and recent/stated experience [32] has shown a high false-positive rate for MPS II newborn screening, attributable to the high frequency of pseudo and pseudo-like alleles in the *IDS* gene [25].

## 5. Conclusions

Newborn screening aims to identify pre-symptomatic patients with treatable diseases that benefit from early diagnosis. The evolution of newborn screening programs due to the availability of new screening technology has pushed the boundaries of newborn screening to not only include identification of babies with ambiguous and non-classical conditions, but also unaffected carriers or pseudodeficiency. The incorporation of post-analytical interpretation and second-tier biomarker testing in the newborn screening process limits detection to high risk patients and avoids the identification of patients with ambiguous findings who face the potential of a lifetime of unnecessary monitoring [16]. With newborn screening programs continually expanding to include more conditions, the need to improve the performance of newborn screening becomes even more important in order to increase testing specificity while decreasing unnecessary parental anxiety and follow up costs due to false-positive results [33,34]. Newborn screening has the capacity to avoid the diagnostic delay and accompanying distress that may be experienced by the patient and family in this scenario, allowing for early identification and timely implementation of treatment.

## Supplementary Materials

The following are available online at https://www.mdpi.com/2409-515X/6/1/10/s1, Table S1: Characteristics of *IDUA* Variants Observed in Patient Cohort.
